# Perceptions and effects of COVID-19 related information in Denmark and Sweden – a web-based survey about COVID-19 and social media

**DOI:** 10.1007/s10389-021-01539-5

**Published:** 2021-04-26

**Authors:** Sigrid Stjernswärd, Anna-Karin Ivert, Stinne Glasdam

**Affiliations:** 1 Department of Health Sciences, Faculty of Medicine, Lund University, Margaretavägen 1 B, S-222 41 Lund, Sweden; 2 Department of Criminology, Malmö University, Jan Waldenströms gata 25, S-214 28 Malmö, Sweden; 3 Integrative Health Research, Department of Health Sciences, Faculty of Medicine, Lund University, Margaretavägen 1 B, S-222 41 Lund, Sweden

**Keywords:** COVID-19 information, Psychosocial effects, Social media, Survey, Denmark, Sweden

## Abstract

**Aim:**

Extensive COVID-19 information can generate information overload and confusion. Denmark and Sweden adopted different COVID-19 management strategies.

**Aim:**

This study aimed to compare search strategies, perceptions and effects of COVID-19 information, in general and specifically in social media, in residents in Denmark and Sweden.

**Subject and methods:**

Quantitative data from a sample of respondents (*n* = 616) from Denmark and Sweden on an international web-based survey was analysed using descriptive and analytical statistics.

**Results:**

The results showed similarities between the countries regarding preferred and trusted information sources, use of (social) media, and psychosocial and behavioural effects of such information. Traditional media and social media were frequently used for COVID-19 information. Especially health authorities and researchers were trusted sources, representing the dominant medico-political discourse. There were no differences in negative effect and social behaviour. Residents in Denmark experienced significantly more positive effects than residents in Sweden.

**Conclusion:**

Summarily, the study showed similarities and small differences among residents in both countries related to usage patterns, perceptions and effects of COVID-19 information from (social) media, despite diverging strategies.

## Introduction

The COVID-19 pandemic, classified as such by the World Health Organization (WHO) on 11 March 2020 (WHO 2020a), including the international and national strategies to contain the SARS-CoV-2 coronavirus have ample consequences for individual citizens and society at large. It represents a global health emergency (Sohrabi et al. 2020). Several containment and mitigation strategies were adopted to eliminate the virus, contain the spreading of COVID-19, and mitigate the negative consequences of the pandemic (Heymann and Shindo 2020). While containing the virus, such measures nevertheless have substantial practical, emotional, economical and political consequences, such as business disruption and bankruptcy, unemployment, educational interruption, social isolation, and more. Some cities, regions or whole countries were completely or partially locked-down by the end of March 2020, thus being subjected to restrictive decrees (Cheng and Khan 2020; Nygren and Olofsson 2020; Sohrabi et al. 2020), while other countries delayed such decisions or adopted less strict strategies. The United Nations and WHO put together advice and recommendations on multiple fronts (United Nations 2020; WHO 2020b). Nonetheless, each country has its own political strategy to handle the pandemic.

This article focuses on two neighbouring countries that chose differing strategies, namely Sweden and Denmark. Denmark was swift with closing its borders and regulating social behaviour by laws (Regeringen 2020). Sweden’s government and Public Health Agency (PHA) opted for recommendations to its citizens, including social distancing and isolation in case of suspected or actual COVID-19 symptoms, and decided not to endorse the strategy of laws and orders to the people (Franssen 2020; Regeringen och Regeringskansliet 2020). Culture and habits are closely related in Sweden and Denmark, borders have been open for many years, people understand each other’s languages, and the connection between the two populations is often labelled as a ‘Scandinavian fraternity’. Despite their similarities, the two countries’ divergence in terms of COVID-19 strategies awakes curiosity (Franssen 2020), which the following media headline illustrates: ‘*The Nordic divide on coronavirus: Which country has the right strategy?*’ (Orange 2020). Based on Sweden’s and Denmark’s various COVID-19 management strategies, it is important to understand how people perceive their own and other countries’ strategies, and how the media in general and specifically social media support these perceptions and contribute to the circulation of COVID-19 related information during the pandemic. Different narratives on COVID-19 circulate in the media, including social media ((Glasdam and Stjernswärd 2020a), which may affect people’s perceptions and handling of the pandemic. Therefore, it is of interest to explore how and what kinds of COVID-19 related information people consume, and its potential effects on its consumers.

## Background

Worldwide, governments ordered lockdowns of varying magnitude in their respective countries when faced with the COVID-19 pandemic (Cheng and Khan 2020; Euractiv 2020; Sohrabi et al. 2020). Mandated to declare a pandemic’s actuality, the WHO also defines global strategies to manage it (Holmberg 2020). As a response to the COVID-19 pandemic, and based on the International Health Regulations (WHO 2005), the United Nations and WHO set up recommendations and containment and mitigation strategies to prevent the virus’ spread and minimise its negative effects, including the control of entry points (United Nations 2020; WHO 2020a, b, c, d). Whilst up against the current pandemic, with no known treatment or vaccine against COVID-19 (Sohrabi et al. 2020), there were no answers about right or wrong to lean against. Information from authorities, governments and organisations with the mandate to express themselves authoritatively on the matter have functioned as guidance in adopting strategies to manage COVID-19, with some countries endorsing laws and orders to control their citizens to restrain the spread of the virus (Cheng and Khan 2020; Sohrabi et al. 2020) and other countries adopting recommendations to the people as a strategy (Regeringen and Regeringskansliet 2020).

Examples of diverging strategies include the handling of border controls, public gatherings, the opening or closing of educational institutions, shops and restaurants (Cheng and Khan 2020; Sohrabi et al. 2020). Denmark closed its borders on 14 March and implemented strict measures to contain the virus by laws. Educational institutions, institutions for children (with an open emergency preparedness) and public workplaces closed for physical attendance on 12–16 March. Public employees were to work from home excepting those with a critical function, e.g. healthcare professionals and the police (Altinget, 2020). Also in Sweden, non-essential travels were to be avoided and work from home was recommended. While all schools in many European countries, including Denmark, closed by mid March, Sweden kept its primary schools open. The Swedish Ministry of Education and Research took this decision as a measure to keep persons in essential occupations available for work. The idea was that it would help minimise societal disruptions and ensure the maintenance of crucial functions in society (Utbildningsdepartementet 2020). Upper secondary and higher level schooling, including university, nevertheless went digital as a measure to contain the virus.

In terms of public gatherings, the WHO issued an assessment tool to estimate the risk of contagion, as a strategy to plan/cancel events for risk minimisation (WHO 2020e). There was however no obvious European consensus on the number of people that could meet. In Denmark, the limit of gatherings was set to a maximum of 10 persons from 18 March until 7 June, and to 50 persons from 8 June (Politi 2020) and 100 persons from 7 July (Regeringen 2020; Folketinget 2020). Sweden banned gatherings of more than 50 persons on 29 March (Folkhälsomyndigheten 2020). However, restaurants, gyms and businesses were kept open, although with restrictions. All the while people were recommended to keep a physical distance and stay at home in case of COVID-19 symptoms. So called ‘risk groups’, understood as elderly people and immunocompromised persons, were encouraged to isolate themselves. In short, Sweden’s government did not impose restrictions as severe as its Scandinavian neighbours, and not by law. Its government seems to have relied on the individual citizen’s sense of responsibility and solidarity in terms of (self) protection and virus containment (Nygren and Olofsson 2020; Regeringen och Regeringskansliet 2020).

In the face of this global crisis, which generates fear and anxiety in many people (Manderson and Levine 2020), the different countries’ governments have thus acted diversely. The pandemic has spurred the scientific community to track, map and prevent the spreading of COVID-19 (Gardner et al. 2020; Qi et al. 2020). Individual citizens can be inundated with information, sometimes contradictory and blurred, and face an ‘infodemic’ (Georgiou et al. 2020; Sahoo et al. 2020; WHO 2020c) that can also spread swiftly through social media (Depoux et al. 2020). COVID-19 is a global issue, attracting the collective attention of governments, (inter)national organisations and individuals. When unreliable information, man-made or bot-made, spreads and when individuals share such information, they become potential infectious agents and contribute to the infodemic (Gallotti et al. 2020). Social media platforms pervade many everyday activities, through entertainment, networking and news (Ventola 2014; Vraga et al. 2018), and facilitate the circulation of varied information and opinions (Depoux et al. 2020; Hollowood and Mostrous 2020). Looking at Denmark and Sweden, both countries received media attention. Denmark’s strategy generated headlines such as ‘*Standing out – Denmark’s approach to dealing with the coronavirus pandemic*’, where Denmark’s handling plan was described as *‘most radical’* and its government as *‘among the first to declare a national lockdown and to close down its borders’* following Italy (Stoyanov 2020). The Swedish epidemiologist Tegnell’s quoted reaction in the following title illustrates differences of opinion about the respective countries’ strategies: *‘Closing borders is ridiculous’: the epidemiologist behind Sweden’s controversial coronavirus strategy*. (Paterlini 2020). By sticking out through its choice of handling plan, the Swedish government attracted the eyes of national and international media, where its strategy was at times depicted as a risky game with people’s lives. Media headlines such as *‘Sweden’s “risky” coronavirus containment strategy criticized’* (Limam 2020) illustrate this. Both critical and puzzled voices relating to the countries’ differing strategies were heard, internationally (Olsen 2020; Savage 2020) and nationally (DN Debatt 2020). The Danish and Swedish strategies were also criticised for insufficient COVID-19 testing (Hilstrøm and Reinwald 2020), governmental unpreparedness in the face of an impending pandemic (Tenitskaja 2020) and home isolation of infected individuals (Krog, 2020). Even the defense of national COVID-19 strategies against critique in the media is debated, as this can be interpreted as misdirected nationalism (Pallas 2020).

Overall, Denmark and Sweden represent two COVID-19 management strategies with differences and similarities. These strategies are both praised and criticised in the media, which gives an idea of the apprehension of the self and others, as closely related neighbouring countries. Different narratives on COVID-19 hence circulate in the media (Glasdam and Stjernwärd 2020a), potentially affecting people’s behaviour pertaining to the pandemic, but also their perceptions of how the pandemic is handled by their respective countries’ authorities. Therefore, this article’s aim is to compare search strategies and perceptions of COVID-19 related information, in general and specifically in social media, in residents in Denmark and Sweden. A further aim is to explore the effects of such information on its consumers.

## Methods

The article is based on data from an international web-based survey, consisting of questions constructed specifically for the current study.

### Instrument and data collection

The survey encompassed 29 structured questions about COVID-19 information and social media, including nine socio-demographic questions (e.g. age, sex, country of residence, educational level, employment status, etc.), out of which a selection in line with the study’s aim was the current article’s focus. The current article hence focuses on nine questions, which are described under *Measures*. The questions had multiple choice response alternatives. The survey, with accompanying information, was distributed through a public link on multiple social media platforms. Participants and people coming across the survey were encouraged to share it with their networks for a snowball effect. The survey was available in eight languages, between 7–28 April 2020. It was answered anonymously and took 10+ minutes to complete.

### Participants

In total, 943 participants answered the web-based survey. The only specified inclusion criteria was age (> 18 years). Focus in the current study is on a sub-sample of respondents (*n* = 616) from Denmark (*n* = 312) and Sweden (*n* = 304), which also represented the countries with most respondents. Most participants in the sub-sample were women (75.4%), aged 40–59 (53.4%), (self) employed (72.7%) and highly educated (71.4%). Table 1 provides information about the distribution of sociodemographic variables by country of residence.

**Table 1:** Distribution of sociodemographic variables by country of residence (percentages)

	Total sample (*N* = 616)	Sweden (n = 304)	Denmark (n = 312)
	%	%	%
Gender			
Females	75.4	74.2	76.6
Males	23.8	24.2	23.4
Age			
18–29 years	13.1	6.9	19.2
30–39 years	14.9	19.1	10.9
40–49 years	22.6	27.0	18.3
50–59 years	30.8	31.6	30.1
60–69 years	12.0	11.8	12.2
70 years or older	6.5	3.6	9.3
Educational level			
Upper secondary school or less	15.0	11.1	18.9
Shorter post-secondary education	13.6	11.4	15.6
University education at bachelor level or above	71.4	77.5	65.5
Employment status pre coronavirus			
(Self) Employed	72.7	84.0	61.5
Unemployed	2.1	0.7	3.5
Homemaker	0.8	0.7	1.0
Student	9.5	6.5	12.5
Retired	11.2	6.2	16.0
Unfit for work	1.6	0.7	2.6
Other	1.9	1.0	2.9

### Measures

The use of different *sources of information* was measured by the question *‘Which is (are) your source(s) of information regarding corona?’*. To examine which sources the respondents found reliable, we used the question *‘Which are, according to you, reliable sources of information about corona?’* The respondents could specify one or more sources (Appendix).

To examine *which social media platforms* the respondents used to look for COVID-19 information, we used the statement *‘I look for/at information about corona in social media on the following platforms’*, followed by a presentation of different social media platforms (Appendix). To grasp what *kind of information* the respondents looked for in social media, we used the statement *‘I look for/at this kind of information about corona in social media’*, followed by a presentation of different types of information (Appendix). To examine *from which sources* the respondents searched for COVID-19 information in social media, we used the statement *‘I search for information about corona in social media from’*, followed by a presentation of different types of sources (Appendix). The response alternatives to those three questions were: strongly agree, agree, neither agree nor disagree, disagree, strongly disagree, which were then collapsed into three categories: agree, neither agree nor disagree, and disagree. To estimate how information about COVID-19 in social media affected the respondents, we created four different indexes. The first index measured *if the respondents had experienced any negative effects* related to COVID-19 information on social media and consisted of eight items: I feel sadness; I feel distressed, I get confused; I feel overwhelmed; I get angry, I get frustrated; I get sarcastic about corona; I feel worried about the future. The second index measured *if the respondents had experienced any positive emotions* related to COVID-19 information on social media and consisted of five items: I feel hopeful; I feel that we can learn something from this experience, I feel safe, I become stronger in my faith; I feel confident about the future. The third index measured *if the respondents had changed their social behaviour as a consequence of COVID-19 information on social media* and consisted of six items. I withdraw from being with family; I withdraw from being with friends; I withdraw from being with colleagues/classmates; I withdraw from being with strangers; I prefer not to leave my home; I prefer not to go outdoors. The fourth index measured *how COVID-19 information on social media had affected how the respondents thought about their own and others’ physical health* and consisted of two items: I am more observant of physical symptoms of corona in myself; I am more observant of physical symptoms of corona in those around me. The response alternatives were: strongly agree, agree, neither agree nor disagree, disagree, strongly disagree, and do not know. Before creating the indexes, the answer alternatives were collapsed into three categories: agree (=2), neither agree nor disagree, (=1) and disagree (=0). The answer alternative ‘do not know’, used by 3% of the respondents, was coded as missing. All scales showed good internal consistency for both the Swedish and Danish sample (alpha >.8) with the exception of the positive effect index that had an alpha of .6.

### Analytical strategy

Logistic regression analysis was used to estimate how country of residence is associated with main sources of information about COVID-19 in general and on social media, as well as perceptions of reliability of different sources. To estimate the association between country of residence and what kind of information respondents looked for on social media and from what sources, we applied multinomial logistic regression analysis. Odds ratios (OR) were calculated with 95% confidence intervals (CI). To estimate if respondents in Sweden and Denmark differed in their reports related to whether they were affected by information about COVID-19 in social media, ordinary least square analysis was conducted. Owing to the higher frequency of younger people among the respondents from Denmark, where significant differences between countries were found, age was adjusted for.

### Ethical considerations

The study adhered to the ethical principles of the Declaration of Helsinki (World Medical Association 2013). Participation was voluntary and data was collected anonymously through a web-based, public survey link. Written information about the study, including contact information to the researchers, accompanied the survey. Participants gave their informed consent by ticking an approval box prior to answering the survey. The findings are presented on aggregated group levels.

## Results

The respondents from both Denmark and Sweden reported TV as the most common source of information about COVID-19 (87.8% and 73.2%, respectively), followed by social media in Denmark (71.2%) and newspapers in Sweden (71.2%). As can be seen from Table 2, Danish residents were significantly more likely to use TV (OR = 2.64, CI = 1.73–4.03) and radio (OR = 1.49, CI = 1.06–2.06) than the Swedish residents (OR = 2.64, CI = 1.73–4.03). In Denmark, respondents were also more likely to get information from social media (OR = 2.08, CI = 1.49–2.90), friends (OR = 2.32, CI = 1.59–3.39) and family (OR = 2.16, CI = 1.52–3.07). Swedish residents were significantly more likely to use newspapers than the Danish residents (OR = 070, CI = 0.50–0.98). Adjusting for age did not affect these associations.

**Table 2:** Logistic regression predicting main source of information. Odds ratios (OR) with 95% confidence intervals (CI) in brackets. N = 616 (Sweden n = 304; Denmark n = 312)

	TV	Radio	Employer	Newspapers	Social media	Friends	Family	Colleagues/classmates	Other sources
	OR (CI)	OR (CI)	OR (CI)	OR (CI)	OR (CI)	OR (CI)	OR (CI)	OR (CI)	OR (CI)
Sweden (ref)	1	1	1	1	1	1	1	1	1
Denmark	2.64 (1.73–4.03)	1.49 (1.06–2.06)	1.18 (0.84–1.65)	0.70 (0.50–0.98)	2.08 (1.49–2.90)	2.32 (1.59–3.39)	2.16 (1.52–3.07)	1.30 (0.89–1.91)	0.52 (0.34–0-79)

All over, there were few differences between respondents living in Sweden and Denmark in relation to which sources of information that were perceived as reliable. Respondents from both countries reported TV as the most reliable source of information (Denmark 80. 3% and Sweden 73%). As can be seen from Table 3, results from the logistic regression analysis show that Danish residents were more likely to perceive TV (OR = 1.50, CI = 1.03–2.19) and social media (OR = 1.70, CI = 1.09–2.66) as reliable sources of information in comparison to Swedish residents. Swedish respondents were, on the other hand, more likely to perceive newspapers (OR = 0.71, CI = 0.52–0.98) and ‘other’ sources of information (unspecified) (OR = 0.41, CI = 0.27–0.62) as reliable. Adjusting for age did not affect these associations.

**Table 3:** Logistic regression predicting reliable sources of information. Odds ratios (OR) with 95% confidence intervals (CI) in brackets. *N* = 611 (Sweden *n* = 302; Denmark *n* = 309)

	TV	Radio	Employer	Newspapers	Social media	Friends	Family	Colleagues/ Classmates	None of the above	I do not care	I do not know	Other sources
	OR (CI)	OR (CI)	OR (CI)	OR (CI)	OR (CI)	OR (CI)	OR (CI)	OR (CI)	OR (CI)	OR (CI)	OR (CI)	OR (CI)
Sweden (ref)	1	1	1	1	1	1	1	1	1	1	1	1
Denmark	1.50 (1.03–2.19)	1.01 (0.73–1.38)	0.74 (0.52–1.06)	0.71 (0.52–0.98)	1.70 (1.09–2.66)	1.63 (0.80–3.32)	1.72 (0.90–3.27)	0.98 (0.53–1.84)	1.71 (0.67–4.42)	–	1.80 (0.59–5.42)	0.41 (0.27–0.62)

In both Denmark and Sweden, Facebook was the most frequently used social media platform to obtain information about COVID-19. Almost 50% of the respondents in both countries agreed that they used Facebook for this purpose (data not shown). Approximately 10% in both countries agreed to the proposition that they used Twitter, Instagram, YouTube or LinkedIn to get information about COVID-19.

Overall, the patterns regarding what kind of information about COVID-19 the respondents looked for in social media were similar across Denmark and Sweden (data not shown). The type of information that most respondents in Denmark and Sweden looked for in social media was authoritative facts (71.1% and 73.5%, respectively), world news (67.9% and 69.8%, respectively), medical issues (65.9% and 73.2%, respectively), national political strategies (66.1% and 65.5%, respectively) and international political strategies regarding COVID-19 (61.5% and 63.9%, respectively). The multinomial logistic regression showed that Swedish residents were more likely to agree to the proposition that they searched for ‘music and art’ (OR = 0.57, CI = 0.35–0.93) and ‘jokes’ (OR = 0.51, CI = 0.35–0.75) than the respondents in Denmark. Adjusting for age did not affect these associations.

In both Denmark and Sweden, the respondents were most likely to report that they used social media to search for information from National health boards and other authorities (77.5% and 80.6%, respectively), followed by information from researchers (77.5% and 80.6%, respectively) and the World health organisation (WHO) (67.4% and 66.9%, respectively). Findings from the multinomial logistic regression showed that Danish residents were more likely to agree to the proposition that they used social media to search for information from politicians (OR = 1.93, CI = 1.31–2.84), the pharmaceutical industry (OR = 1.78, CI = 1.17–2.71), healthcare professionals (OR = 2.81, CI = 1.87–4.24), patient organisations (OR = 3.79, CI = 2.37–6.07) and artists (OR = 2.80, CI = 1.43–5.46) than Swedish residents. Adjusting for age did not affect these associations.

There was no significant association between country of residence and the index measuring *negative emotions* from COVID-19 information in social media (Table 4). Looking at the separate items nonetheless shows that respondents in Sweden were more likely to agree to the propositions that COVID-19 information in social media made them feel sadness (58.5% vs 43.2%, *p* = .001) and distress (48.2% vs 16.3%, *p* = .000). Respondents in Denmark were more likely to agree to the propositions that information in social media about corona made them get sarcastic about COVID-19 (15.2% vs 10.2%, *p* = .043) and that they felt worried about the future (50.4% vs 39.9%, p = .043).

**Table 4:** Ordinary least square regression of the association between country of residence and reported effects of information about COVID-19 in social media

	Negative emotions (min: 0, max: 16)		Positive effect (min: 0, max: 10)		Changed social behaviour (min: 0, max:12)		Attention to physical health (min: 0, max:4)	
	*B*	*SE*	*B*	*SE*	*B*	*SE*	*B*	*SE*
Country of residence (Sweden = 0, Denmark =1).	−.231	.382	.789***	.200	.077	.331	−.281*	.121

* = *p* < .05, ** = *p* < .01, *** = *p* < .001

As can be seen from Table 4, there is a positive association between country of residence and the index measuring *positive effect* from COVID-19 information in social media. This indicates that respondents from Denmark to a higher extent experienced a positive effect from information about COVID-19 in social media. Respondents in Denmark were more likely to agree to the propositions that COVID-19 information in social media made them become stronger in their faith (10.4% vs 4.3%, *p* = .000), while respondents in Sweden to a higher degree reported that they agreed to the propositions that people can learn something from this experience (84.8% vs. 72%, *p* = .001) and that they feel confident about the future (49.5% vs 21.9%, *p* = .000).

Furthermore, the analysis showed no association between country of residence and *how information about COVID-19 affected respondents’ social behaviour*. However, differences could be observed in relation to separate items. Respondents living in Denmark were more likely than respondents living in Sweden to agree to the proposition that they withdraw from being with colleagues and classmates (56.5% vs 46%, *p* = .044) and that they withdraw from being with strangers (70.8% vs 65.6%, *p* = .046).

Finally, respondents living in Sweden scored higher on the index measuring *how they think about their own and others’ physical health*, indicating that COVID-19 information in social media to a larger extent affected how they thought about their physical health.

## Discussion

Despite Denmark’s and Sweden’s differing management strategies regarding the pandemic, the current findings show both similarities and differences between the two countries’ respondents regarding their reported uses of sources of COVID-19 information, trust in these sources, and psychosocial and behavioural effects of COVID-19 related information.

The results show a general trust in information stemming from parties viewed as authoritative by the respective countries’ residents, i.e. (inter)national authorities, national health boards, researchers and health experts. This could be interpreted as trust in the strategy of the country where the respondents live. Denmark and Sweden are welfare states. Although the welfare state is a relatively new historical phenomenon, it has a long pre-history and is an essential part of the Danish and Swedish cultural heritage (Raffnsøe 2008). It has become the single most cohesive element in the social fabric, being based on a social contract that is constantly reproduced in and through the welfare society, its institutions and the various forms of social interaction it imbues. The welfare state’s idea is to care for all and everyone (Raffnsøe 2008). The vast majority of people living in Denmark and Sweden grew up in their respective countries (Bjerre et al. 2019); however, it is unknown whether this is also the case for this study’s respondents. People who grow up in a specific country are nevertheless primarily socialised to understand the country’s explicit and implicit rules and logics, which is why it often seems ‘natural’ to them to accept the cultural and political premises that apply in that country (Berger and Luckmann 1966). Vallgårda (2007) shows that in several social and political issues, politicians in Denmark and Sweden problematised social inequalities in health differently, despite many similarities between the two welfare states. There were differences in timing, reasons for dealing with the issue, descriptions, explanations and suggested solutions regarding social inequalities. The policies chosen to address social inequalities in health follow the same pattern as the general public health policies in the two countries (Vallgårda 2007). In other words, the respondents from Denmark and Sweden are historically and culturally used to different strategies regarding health, and most of them trust their own country, the national health authorities and the politicians in their chosen strategies regarding health, also when it comes to the COVID-19 pandemic. According to the philosopher L.-H. Schmidt, citizens of Denmark and Sweden have basic confidence in their governments acting in the best interests of all (Gerstenberg 2020). The current results can be interpreted as pointing in the same direction. The citizens believe that when the state says ‘this is how we should do’ and there is minimal opposition, they can feel safe. Citizens play by the rules and stand together. They are critical, but they are confident in times of crisis (Gerstenberg 2020). An international study including 14 countries with advanced economies actually shows that most people approve of their respective national response to COVID-19. Denmark topped the list, with 95% of the public approving of the country’s response to the pandemic, in Sweden the figure was 71% (Devlin and Connaughton 2020). Country specific timing, as well as transparency and trust are critical for compliant response to mitigation advice among the people, affecting its impact (Ebrahim et al. 2020; Balog-Way and McComas 2020). Nonetheless, the uncertainty associated with the COVID-19 pandemic and its rapid spread represents a challenge for building trust (Balog-Way and McComas, 2020). Even mixed messages and the pace of change in message contents from leading risk communicators, for example, in terms of wearing facemasks or not, challenge trust building among the people (Balog-Way and McComas 2020). Transparency is also critical to build trust, nevertheless transparency can have the opposite effect, for instance, through bombardments with mixed messages or when the receivers cannot properly process and use the information (Balog-Way and McComas 2020; Heald 2006). The current findings however indicate high levels of trust from residents of both Denmark and Sweden in the respective countries’ authorities when it comes to COVID-19 related information. These assumptions must nevertheless be interpreted with caution, not the least as most participants in the current study were highly educated, which per se may affect both access to information and the ability to assess it critically. Furthermore, the limited sample may not be representative of the wider populations of Sweden and Denmark, respectively. Furthermore, the current results can not illuminate whether and how trust in general COVID-19 related information subsequently affects the respondents’ perceptions of their respective countries’ handling strategies. Neither can they throw light on how these strategies potentially affect people’s behaviour, e.g. in comparison to how the consumed information affects them. These questions rather invite further research on the subject.

In line with other studies (Casero-Ripollés 2020), the results showed that residents of both Sweden and Denmark used traditional media such as TV, radio and newspapers, as main information sources during the COVID-19 crisis, although changes in news consumption prior to and during the COVID-19 pandemic were not assessed. Furthermore, social media platforms represented common channels to look for COVID-19 related information during the pandemic for residents in both countries, without relation to age. The results further indicate that information retrieved from social media platforms, in both countries’ residents, is reported as mostly stemming from organisations and parties in society deemed as authoritative, i.e. national health boards and other authorities, researchers and the WHO. This is also where the dominant medico-political discourse on COVID-19 originates from. The WHO has a mandate to express itself authoritatively on the subject and outlines global strategies to manage the pandemic (Holmberg 2020). Just like the WHO, government agencies and other authorities have established their presence in social media for strategic dissemination of information during crises (O’Brien et al. 2020). The current findings partly support this by showing that social media are commonly used to search for COVID-19 information. The results also indicate that most residents in both Sweden and Denmark view such organisations as reliable sources of information, although the countries have adopted differing COVID-19 strategies. Nonetheless, residents in Denmark were more likely to use social media to search for information from additional sources, including politicians, the pharmaceutical industry, healthcare professionals, patient organisations, artists and religion/faith representatives.

In terms of effects of COVID-19 information on social behaviour, the findings showed that respondents in both countries to a certain point withdrew from socialising with colleagues, friends and strangers. Denmark’s more restrictive strategies may impede on people’s possibilities and proclivity to socialise, as compared to the Swedish government’s recommendations and trust in people’s sense of moral responsibility to follow the new behavioural rules. Nevertheless, such restrictions may also ignite resistance (Foucault, 1995) and new ways of socialising (Glasdam and Stjernswärd 2020b). An international study showed that the pandemic seems to have had a divisive effect on people’s sense of national unity, with 72% in Denmark compared to 58% in Sweden feeling that the country was more united after than before the outbreak (Devlin and Connaughton 2020). Relaxation rules, together with people’s less strict adherence to practices such as hand-washing and social distancing, are likely to cause tensions. Moralisation of such behaviour risks creating interactional trouble, with people valuing each other as do-gooders versus wrong-doers (Prosser et al. 2020). This, together with trust or lack thereof in the authorities’ strategies, may hence also affect how people choose or not to socialise with consideration taken to authoritative socialisation advice. As implied by the COVID-19 ‘infodemic’, citizens can be faced with information overload (Ahmed 2020; Gallotti et al. 2020; Kulkarni et al. 2020), encompassing accurate and timely information (O’Brien et al. 2020), but also what those behind the dominant medico-political discourse would classify as dis- and misinformation (Depoux et al. 2020; Hollowood and Mostrous 2020). The rapid spread of information through social media certainly contributes to this phenomenon, and may cause confusion and panic in some people (de Vries et al., 2018; Depoux et al., 2020). Discerning true from false, valid information from noise, requires time and skills, and depends on which perspective information is assessed from (Georgiou et al. 2020; Sahoo et al. 2020). Repeated exposure to COVID-19 news, not the least through social media, can engender stress, anxiety and depression in some people, which is why limitations in terms of exposure to such information can be suggested (Ni et al., 2020). Nonetheless, digital platforms including social media have facilitated the maintenance of social contact with family, friends and colleagues, contributing to (partial) continuity of work/education and preventing total isolation, but also representing a risk of overburdening through the fusion of, for example, home and workplace (Fuchs 2020). Paying attention to physical symptoms, in the self and others, is maybe evident during a pandemic. Taken to its extreme, it may nonetheless generate exaggerated attention to physical symptoms, and also demonisation and polarisation when this attention, and potential moral judgment (Prosser et al. 2020), is directed at others.

As to the methods, anonymous responses to the survey prevents bias to please. The findings must nonetheless be interpreted with the sample’s sociodemographic background in mind. Most participants were middle-aged, highly educated women, although the sample had a higher representation of younger people among residents from Denmark as compared to Sweden. Furthermore, the current sample may not be representative of the wider populations of Denmark and Sweden respectively, especially since data collection online entails the risks of self-selected and biased samples (Wright 2005). The snowball sampling strategy may also explain lack of diversity in terms of the sample’s sociodemographic background. Altogether, this limits the results’ generalisability. Another limitation is that the respondents’ nationality is unknown, which could have significance for the answers. In spite of these limitations, the current results illuminate perceptions of information related to COVID-19, in general and specifically in social media, in residents from Denmark and Sweden, and how these perceptions affected them at relatively early stages of the pandemic.

## Conclusions

Denmark and Sweden adopted different management, political strategies to handle the COVID-19 pandemic in their respective countries. The results showed both similarities and differences in the respective country residents’ patterns regarding preferred and trusted sources for COVID-19 related information, including psychosocial and behavioural effects of the latter. In both countries, similar sources reached through both traditional media and social media were used to search for such information. Similarities were also found in terms of trusted information sources, which included authorities, the WHO, researchers and healthcare professionals, also when searching for information through social media. Nonetheless, residents in Denmark looked for significantly more different kinds of information on social media compared to residents in Sweden. As for the effects of such information, there were both similarities and differences, especially on separate index items pertaining to positive effects of information, attention to physical symptoms, and to socialising with people outside the family. Faced with a COVID-19 ‘infodemic’, people can find themselves inundated with a diversity of information and opinions from multiple sources and channels, including social media. At the same time, social media functions as a way to socialise in a historic time where social distancing is the mantra and a social, collective expectation. In sum, the study found many similarities and small differences among residents in Denmark and Sweden regarding preferred and trusted sources of COVID-19 information in the pandemic despite the different national main strategies. However, this study calls for further research on people in Sweden and Denmark to understand the complexity of the neighbourhood, the Scandinavian fraternity, and all the inherent cultural similarities and differences in the face of a pandemic. Further research is also necessary regarding how people in Denmark and Sweden, with a greater variation of socio-demographic backgrounds, perceive and handle COVID-19 related information, including its effects on individuals’ psychosocial health and behaviour.

## Data Availability

The authors elect to not share data for confidentiality reasons.

## Appendix

Excerpt of survey questions
